# Ebola outbreak in Guinea, 2021: Clinical care of patients with Ebola virus disease

**DOI:** 10.4102/sajid.v38i1.454

**Published:** 2023-01-31

**Authors:** Boyo C. Pare, Alseny M. Camara, Aminata Camara, Moussa Kourouma, Koivogui Enogo, Mohammed S. Camara, Laurent Akilimali, Sayadi Sani, Eric Barte de Sainte Fare, Papys Lame, Nicolas Mouly, Marta Lado Castro-Rial, Billy Sivahera, Mahamoud S. Cherif, Abdoul H. Beavogui, Dally Muamba, Joachim B. Tamba, Barry Moumié, Richard Kojan, Hans-Joerg Lang

**Affiliations:** 1 Alliance for International Medical Action (ALIMA), Dakar, Senegal; 2 Ministry of Health, Agence Nationale de Sécurité Sanitaire, N’zérékoré, Guinea; 3 Ministry of Health, Hôpital Régionale de N’zérékoré, N’zérékoré, Guinea; 4 World Health Organization (WHO), Geneva, Switzerland; 5 Centre National de Formation et de Recherche en Santé Rural de Maferinyah, Maferenya, Guinea; 6 Witten/Herdecke- University, Global Child Health, Witten, Germany

**Keywords:** Zaire Ebolavirus disease essential emergency and critical care, referral pathways, Ebola-specific monoclonal antibodies, Ebola vaccination, Lassa virus disease

## Abstract

**Background:**

Experience from the Zaire Ebolavirus epidemic in the eastern Democratic Republic of the Congo (2018–2020) demonstrates that early initiation of essential critical care and administration of Zaire Ebolavirus specific monoclonal antibodies may be associated with improved outcomes among patients with Ebola virus disease (EVD).

**Objectives:**

This series describes 13 EVD patients and 276 patients with suspected EVD treated during a Zaire Ebolavirus outbreak in Guinea in 2021.

**Method:**

Patients with confirmed or suspected EVD were treated in two Ebola treatment centres (ETC) in the region of N’zérékoré. Data were reviewed from all patients with suspected or confirmed EVD hospitalised in these two ETCs during the outbreak (14 February 2021 – 19 June 2021). Ebola-specific monoclonal antibodies, were available 2 weeks after onset of the outbreak.

**Results:**

Nine of the 13 EVD patients (age range: 22–70 years) survived. The four EVD patients who died, including one pregnant woman, presented with multi-organ dysfunction and died within 48 h of admission. All eight patients who received Ebola-specific monoclonal antibodies survived. Four of the 13 EVD patients were health workers. Improvement of ETC design facilitated implementation of WHO-recommended ‘optimized supportive care for EVD’. In this context, pragmatic clinical training was integrated in routine ETC activities. Initial clinical manifestations of 13 confirmed EVD patients were similar to those of 276 patients with suspected, but subsequently non confirmed EVD. These patients suffered from other acute infections (e.g. malaria in 183 of 276 patients; 66%). Five of the 276 patients with suspected EVD died. One of these five patients had Lassa virus disease and a coronavirus disease 2019 (COVID-19) co-infection.

**Conclusion:**

Multidisciplinary outbreak response teams can rapidly optimise ETC design. Trained clinical teams can provide WHO-recommended optimised supportive care, including safe administration of Ebola-specific monoclonal antibodies. Pragmatic training in essential critical care can be integrated in routine ETC activities.

**Contribution:**

This article describes clinical realities associated with implementation of WHO-recommended standards of ‘optimized supportive care’ and administration of Ebola virus specific treatments. In this context, the importance of essential design principles of ETCs is underlined, which allow continuous visual contact and verbal interaction of health workers and families with their patients. Elements that may contribute to further quality of care improvements for patients with confirmed or suspected EVD are discussed.

## Introduction

The most devastating Ebola epidemic to date occurred in West Africa between 2013 and 2016. More than 28 000 developed Zaire ebolavirus disease (EVD), and more than 11 000 died.^1,2,3^ Around 2500 of these deaths occurred in Guinea, the starting point of this epidemic.^1,2,3^

Since 2016, further Ebola outbreaks have occurred in the Democratic Republic of the Congo (DRC) and now Uganda.^1^ During this period, substantial advances have been made in introducing Ebola vaccines and improving the level of clinical care for EVD patients.^1,4,5,6,7,8^ Treatment provided in Ebola treatment centres (ETCs)^9^ during the Zaire ebolavirus epidemic in the eastern DRC (2018–2020) followed principles of ‘essential emergency and critical care (EECC)’ outlined in the World Health Organization (WHO) guidelines (‘optimized supportive care’).^4,7,8,9,10^ Additionally, in a randomised controlled trial (RCT; *Pamoja Tulinde Maisha* [PALM] trial) conducted in the DRC (2018–2019), administration of Ebola-specific monoclonal antibodies (mAb114, Ebanga; REGN-EB3, Inmazeb) was associated with lower mortality.^6^ Ebola virus disease patients presenting to an ETC early in their disease process with low viral load (VL) benefited from a care package of essential critical care and Ebola-specific treatment.^4,6,7^ Survival rates above 80% were reported in these patients.^6^

Optimised design of ETCs allows visibility of patients from the low-risk zone, which enables continuous monitoring and improved communication between clinical teams working in low- and high-risk zones. This configuration facilitates provision of essential critical care and psychological support for patients with confirmed or suspected EVD.^5,9^

At the end of the Ebola epidemic in West Africa (2013–2016), vaccinations were introduced as an essential part of a comprehensive outbreak response strategy combining preventive and therapeutic elements.^1,11^ All aspects of outbreak management are based on intensive community engagement.^1^

In February 2021, Guinea declared a further Zaire ebolavirus outbreak in a region (N’zérékoré) previously affected during the epidemic in 2013–2016.^1,2,12,13^ By the end of the outbreak in June 2021, 23 Ebola virus infections were reported (16 confirmed and 7 probable cases). Of these 23 patients, 11 survived and 12 died.^12,13^

In this series we describe 13 patients with confirmed EVD and 276 patients with suspected EVD treated in two ETCs, and their clinical outcomes.

## Methods

**Figure 1:**
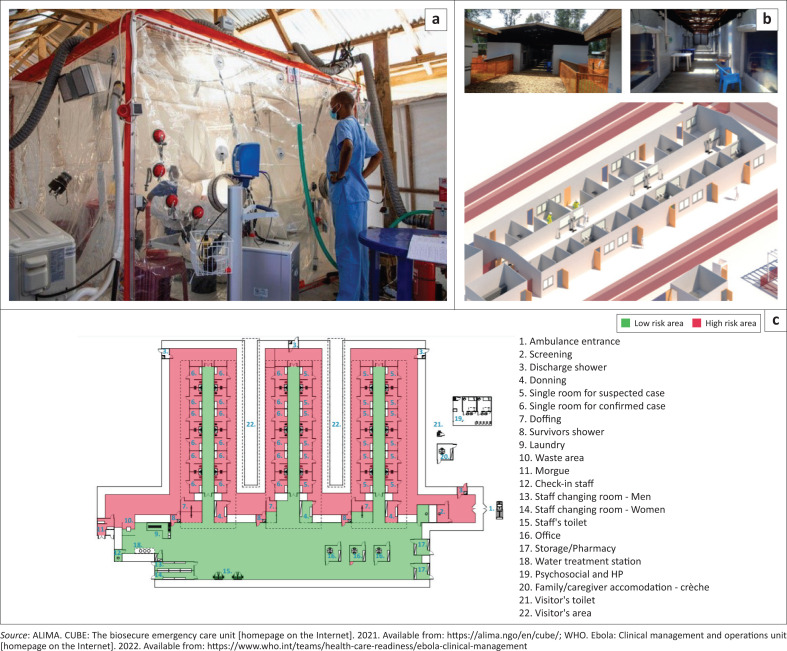
The setup of a modern-design Ebola treatment centre (ETC) and the *chambre d’urgence biosécurisée pour épidémies* or biosecure emergency rooms for epidemics (CUBE). Patients with confirmed or suspected Ebola virus disease are treated in single compartments (b, c). Each patient is visible from the low-risk zone. The CUBE (a) is of particular benefit for the treatment of critically ill patients and can be integrated rapidly in ETC setups as well as patient circuits of general health facilities.Pictures (b, c) from the DRC (2018–2020), with permission to print from Luca Fontana and Michele DiMarco (WHO).

The N’zérékoré region has a population of around 480 000 inhabitants, distributed over 11 subprefectures.^14^ At the end and after the Ebola epidemic in 2014–2016, centres de traitement épidémiologiques (CT_Epi) were established in several regions in Guinea and were designed to provide care for patients with highly infectious diseases (e.g. Ebola, Lassa, Cholera, COVID-19). As part of the outbreak response, clinical care for patients with confirmed or suspected EVD was provided in two Centres de Traitement Épidémiologique (CT-Epi or ETC), led by the Agence Nationale de Sécurité Sanitaire (ANSS), the outbreak response agency of the Guinean Ministry of Health (MOH).^15^ These two ETCs were located in two of the four subprefectures directly affected by the Ebola outbreak. Clinical care in ETCs was supported by the nongovernmental organisation Alliance for International Medical Action (ALIMA) and the WHO.^12,16^ Both CT-Epis or ETCs needed some adaptation to allow adequate care for patients with highly infectious diseases (HID; Figure 1).^5,9^

In this series, data are reviewed from all patients with suspected or confirmed EVD hospitalised in the ETCs of N’zérékoré and Gouécké during the outbreak period (14 February 2021 – 19 June 2021).

### Authorisation for publication

Authorisation for publication of data was given by Guinean health authorities.^15^ REGENERON-EB3 (REGN-EB3) was administered following WHO recommendations for treatment of Zaire ebolavirus disease.^1,17,18^ Care provided in ETCs followed international critical care strategies adapted to contexts of an Ebola outbreak in resource-limited settings.^4,10,19,20^ A pragmatic training programme in essential critical care integrated in routine ETC activities was designed to contribute to general capacity building.

### Set-up of Ebola treatment centres and description of level of care provided

The ETC in the urban setting of N’zérékoré acted as a referral unit for all confirmed EVD patients, critically ill suspected cases and nonsevere direct admissions. This ETC had a capacity of 34 beds and three ‘Chambre d’Urgence Biosécurisée pour Epidémies [*CUBE; biosecure emergency r ooms for epidemics*]’ (see Figure 1).^4,5^ If necessary, two more CUBEs could have been set up rapidly to expand the critical care zone of the ETC.

The ETC located in Gouécké (54 km from N’zérékoré) was set up for the care of patients with suspected EVD. This unit had a capacity of 10 beds and two CUBEs. Competencies of the ETC team in Gouécké to provide prereferral management for critically ill patients with suspected EVD were strengthened. Referrals were conducted with ambulances, allowing basic care during transfer while maintaining recommended infection prevention measures.^1^ Competencies in patient transfer were strengthened. Communication channels between referring and receiving units as well as the outbreak coordination team were improved.

Newly designed ETCs suggested by WHO and partners allow continuous observation of confirmed or suspected EVD patients from the low-risk zone, either via simple windows (e.g. plexiglass) or via CUBEs (Figure 1).^5,9^ This setup allows constant interaction between patients, health workers and relatives. To prevent cross-infection, patients are placed in self-contained rooms (with toilet and shower). Additionally, patients with suspected or confirmed EVD are treated in separate sections of the ETC.

The CUBE is an innovative single treatment room for patients with HIDs, which was used extensively by ALIMA and partners in three ETCs during the Ebola epidemic in the DRC (2018–2020).^5,16^ These CUBEs can be deployed and integrated rapidly in ETCs as well as patient circuits of general health facilities to establish critical care zones during epidemics.^5,9^ The device allows continuous surveillance of critically ill patients with HIDs, while following biosecurity recommendations.^1,5^ Certain clinical interventions can be conducted from the low-risk zone, for example, vital sign monitoring, O_2_ administration, point-of-care ultrasound (POCUS) and adaptation of infusion settings.^5,9^ Clinical teams in the low-risk zone can coordinate activities with health workers entering the high-risk zone with personal protection equipment (PPE).^5,9^

**Figure 2:**
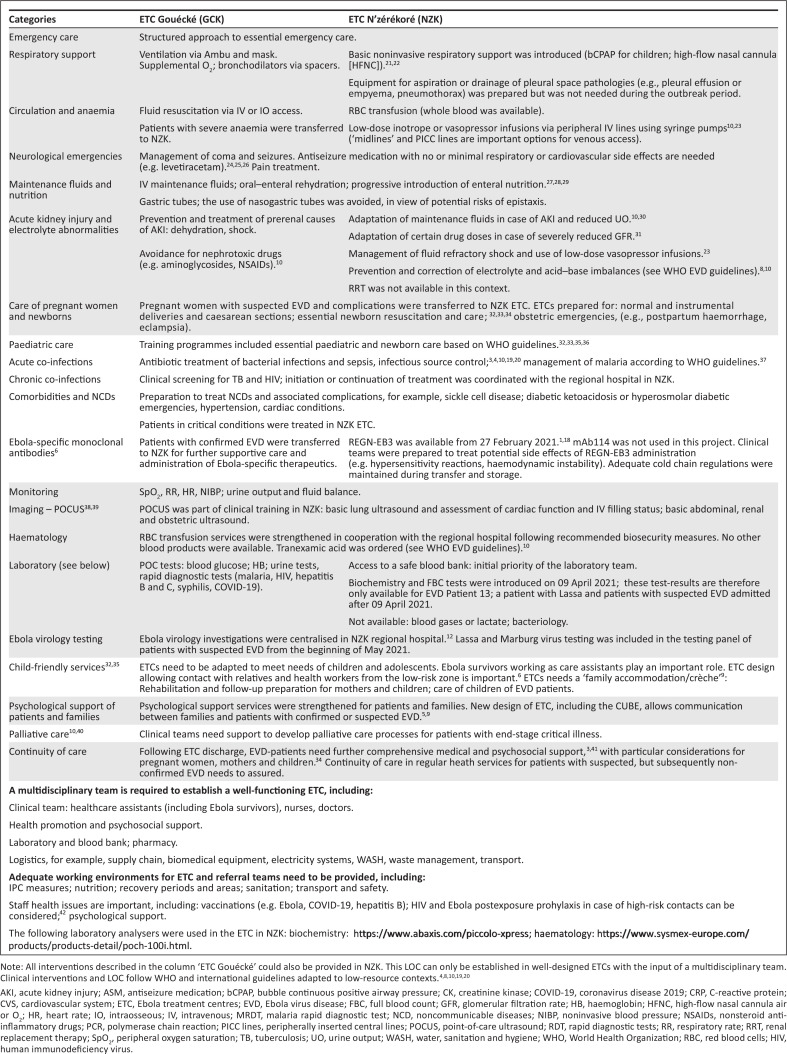
Simplified description of level of care provided in Ebola treatment centres in N’zérékoré (NZK) and Gouécké (Guinea 2021).

Figure 2 describes the level of care (LOC) provided in the two ETCs in the N’zérékoré region, following WHO-EVD guidelines and international recommendations for context-adapted critical care.^4,8,10,19,20^ A similar LOC was established in ETCs during the Ebola outbreak in the DRC (2018–2020).^7,16^ Not all elements of this ‘care package’ could be established from the beginning of the outbreak in Guinea.

### Blood bank and laboratory services

Specific polymerase chain reaction (PCR) testing and VL analysis for Ebola virus were established in the regional hospital in N’zérékoré.^12^ On-demand coronavirus disease 2019 (COVID-19) PCR testing could be performed. Lassa and Marburg virus testing was included in the testing panel of patients with suspected EVD from the beginning of May 2021.^43,44^

The initial priority of the ETC laboratory team was to strengthen safe blood transfusion processes in collaboration with national transfusion services and the regional hospital. Later, during the Ebola outbreak, a small ETC laboratory was established using a modified CUBE, allowing rapid processing of blood samples (e.g. biochemistry, haemogram) by technicians working in the low-risk zone, while respecting biosecurity recommendations.

### Capacity building

A training programme supported by a WHO advisor and a paediatrician with critical care experience (20 March 2021 to 20 April 2021) was integrated in routine ETC activities in N’zérékoré and Gouécké. The instructors (H.J.L. and M.L.) gained experience during previous Ebola outbreaks. Training materials were developed during the Ebola epidemic in the DRC (2018–2020) and reviewed for the outbreak in Guinea by specialists associated with the WHO, ALIMA and international partners (Several authors [R.K., H.J.L., M.L., A.M.C.] are part of the WHO-EVD guideline development team).^18^ Training content followed principles of essential critical care adapted to resource-limited contexts tailored to characteristics of EVD.^4,8,10^ Treatment of specific patient populations was integrated in the training, for example, obstetric emergencies, newborn and paediatric care. Training methods included ‘on-the-job’ coaching, tutorials, skills training, case discussions and clinical simulations integrating utilisation of the CUBE.^5,9^

### Data collection and data analysis

Data were extracted from patient files, for example, gender, age, clinical presentation, treatment, clinical progress and available laboratory results. Clinical details documented in a database were categorised to describe organ dysfunction, potential acute or chronic co-infections (e.g. malaria, human immunodeficiency virus [HIV], tuberculosis [TB]) or underlying noncommunicable diseases (NCDs). The WHO EVD case definition served as a basis for clinical screening and symptom assessment.^45^

Stata 12 (StataCorp LLC, College Station, Texas, United States) was used for all descriptive analyses. In view of the small number of patients, no statistical analysis comparisons were performed.

### Ethical considerations

This study describes a series of patients with confirmed or suspected Ebola virus disease (EVD). Described clinical management follows the WHO recommendations and international guidelines (important guidelines and recommendations are referenced). This is not an interventional study. A formal ethics committee was therefore not required.

The Guinean Ministry of Health (MOH) authorised the use of Ebola-specific monoclonal antibodies as recommended by the WHO, based on the outcomes of a randomised controlled trial (RCT) published in 2019 (relevant sources are referenced in the article).

## Results

### Sociodemographic description of patients

During the study period, 13 patients with EVD and 276 patients with suspected EVD were treated in two ETCs in the N’zérékoré region (Table 1). During the outbreak, 2 of the 16 confirmed EVD patients were not admitted to an ETC (one survivor and one death).^12,13^ One EVD patient was treated in an ETC in Conakry and survived.^12,13^ Of the 13 described EVD patients, the age range was 22–70 years; seven were female, including one pregnant woman. Four (31%) EVD patients were health workers.

**TABLE 1:** Sociodemographic description of Ebola virus disease patients and patients with suspected Ebola virus disease.

Population	Confirmed (*N* = 13)			Suspected† (*N* = 276)		
	*n*	%	Mean age ± s.d. (years)	*n*	%	Mean age ± s.d. (years)
**Gender**	13	-	-	276	-	-
Female	7	54.00	-	135	49.00	-
Pregnant patients	1	8.00	-	7	3.00	-
Male	6	46.00	-	141	51.00	-
**Age ranges**	13	-	-	276	-	-
< 1 year	0	0.00	-	4	1.00	-
1–5	0	0.00	-	43	16.00	-
5–15	0	0.00	-	59	21.00	-
15–25	1	8.00	-	48	17.00	-
25–60	9	69.00	-	99	36.00	-
> 60 years	3	23.00	-	23	8.00	-
**Profession**	13	-	45.6 ± 16	239	-	26.3 ± 21
Health worker	4	31.00	-	17	7.00	-
Student	0	0.00	-	62	26.00	-
Housewife or husband	5	39.00	-	37	15.00	-
Farmer	0	0.00	-	14	6.00	-
Others	4	31.00	-	109	46.00	-

s.d., standard deviation; IQR, interquartile range.

† , EVD among patients with suspected disease was ruled out after two negative PCR tests within 48 h.

### Clinical presentation of patients, treatment and outcomes

Among 10 of the 13 EVD patients, the following nonspecific symptoms were reported by the outbreak transfer team prior to ETC admission: fever, headache, diarrhoea and abdominal pain. Five EVD patients had a positive malaria rapid diagnostic test (MRDT; Table 2).

**TABLE 2:** Clinical signs and malaria rapid diagnostic test results in Ebola virus disease patients and patients with suspected Ebola virus disease.

Clinical signs and MRDT test results	Confirmed (*N* = 13)		Suspected (*N* = 276)	
	*n*	%	*n*	%
**MRDT**	13	-	276	-
MRDT +	5	38	183	66
MRDT −	8	62	93	34
**Respiratory system**				
Cough	1	8	90	33
Breathing difficulties	3	23	40	14
Sore throat	3	23	13	5
**Circulation and haemostasis**				
Signs of bleeding†	5†	38	7	3
**Gastro-intestinal manifestations**				
Nausea and vomiting	6	46	119	43
Diarrheal	10	77	42	15
Abdominal pain	10	77	143	52
Jaundice	0	0	4	1
**Neurological manifestations**				
Altered level of consciousness	1	8	4	1
Confusion or disorientation	3	23	10	4
Swallowing difficulties	3	23	15	5
Headaches	10	77	177	64
General fatigue	8	62	185	67
**Musculoskeletal system**				
Thoracic pain	2	15	45	16
Muscle pain	5	38	84	30
Joint pain	6	46	76	28
**Skin and eyes**				
Rash or other skin manifestations	0	0	14	5
Conjunctivitis	0	0	3	1
**Others**				
Fever	10	77	210	76
Loss of appetite or anorexia	9	69	160	58
Hiccups	2	15	5	1

MRDT, malaria rapid diagnostic test.

† , Signs of bleeding, for example, epistaxis, bleeding from oral mucosa, gastro-intestinal bleeding, bleeding from injection sites.

Note: Epidemiological context: confirmed EVD patients: nine EVD patients had close contact with confirmed Ebola cases; four EVD patients had close contact with ‘probable cases’. Patients with suspected EVD: 15 patients had contact with EVD patients. These data may have limited accuracy as this information depends on verbal communication with patients or relatives during a stressful admission process to an ETC. Transfer of patients with suspected but subsequently nonconfirmed EVD: five of the 271 surviving patients with suspected EVD were transferred to other health facilities after Ebola virus infection was excluded. One of these five patients was transferred to a COVID-19 treatment centre. Only three of the 13 EVD patients (23%) had signs of severe haemorrhage potentially requiring transfusion and further blood products (EVD number: 1; 9; 11 [Online Appendix 1]). All three of these patients presented with multi-organ dysfunction and died within 48 h of ETC arrival. Viral load was only available in one of these three patients with severe haemorrhage (nucleoprotein cycle threshold [NP-CT]:22; EVD 9).

Among the 276 patients with suspected but nonconfirmed EVD, the most frequent symptoms recorded by the transfer team were: fever (76%), fatigue (67%), headache (64%) and loss of appetite (58%). Sixty-six percent of suspected cases had a positive MRDT. Diarrhoea was recorded in only 15%.

Documentation of time between onset of symptoms and ETC admission was challenging because of its dependence on communication with patients or relatives during a potentially stressful admission process. A delay of up to three days was documented in two nonsurviving EVD patients (Table 3 and Online Appendix 1^48^). One of these patients was pregnant (32–34 weeks’ gestation) and deteriorated rapidly after ETC admission.

**TABLE 3:** Characteristics of patients admitted to the Ebola treatment centres in N’zérékoré and Gouécké (confirmed and suspected Ebola virus disease).

Variable	Confirmed (*N* = 13)					Suspected (*N* = 276)				
	*n*	%	Median	IQR	Mean ± s.d	*n*	%	Median	IQR	Mean ± s.d
**Duration between onset of symptoms and admission (days)**	-	-	5	3–9	-	-	-	3	2–6	-
**Duration between onset of symptoms and ETC-admission (days)**	13	-	-	-	-	275†	-	-	-	-
Admission < 3 days	3 (2 deaths‡)	23	-	-	-	113	41	-	-	-
Admission between 3 to 7 days	6 (1 death)	46	-	-	-	123	45	-	-	-
Admission > 7 days	4 (1 death)	31	-	-	-	39	14	-	-	-
**Ebola vaccination (> 14 days before onset of symptoms)¶**										
**Clinical outcome**	13	-	-	-	-	276	-	-	-	-
Survival and discharge	9	69	-	-	-	271	98	-	-	-
**Death during admission**	4	31	-	-	-	5††	2	-	-	-
Time between admission and death among nonsurvivors (days)	4	-	-	-	-	5	-	-	-	-
< 24 h	2	50	-	-	-	3	60	-	-	-
24 h – 48 h	2	50	-	-	-	2	40	-	-	-
> 48 h	0	0	-	-	-	-	0	-	-	-
Cycle threshold (CT) for Zaire ebolavirus PCR at admission: NP-CT ≤ 22‡‡ *(CT results are available for eight patients)*	3 (2 deaths)	-	-	-	-	-	-	-	-	-
Ebola viral load at admission: NP-CT > 22‡‡	5 (0 deaths)	-	-	-	-	-	-	-	-	-
Patients who received REGN-EB3 (Note, no patient received mAb 114 during the study period)††	8 (0 deaths)	-	-	-	-	-	-	-	-	-
Number of patients not received REGN-EB3	5 (4 deaths)	-	-	-	-	-	-	-	-	-
Time between admission to start of REGN-EB3 (days)§§	-	-	-	-	4.3 ± 2.3	-	-	-	-	N/A
Time between admission to start of REGN-EB3 (days)§§	-	-	4.5	2.5–5.5	-	-	-	0	0–0	-
Length of stay in the CT-EPI (days)	-	-	11	2–21	-	-	-	2	2–3	-

Note: 6% (16/276) patients with suspected but nonconfirmed EVD required RBC transfusions.

CT, cycle threshold; ETC, Ebola treatment centres; GCK, Gouécké; IQR, interquartile ranges; NP, nucleoprotein; NZK, N’zérékoré; PCR, polymerase chain reaction; s.d., standard deviation; NT-CT, nucleoprotein-cycle threshold; RBC red blood cells; RBC, red blood cells.

† , For one patient the time of onset of symptoms is unknown;

‡ , One of these two patients was pregnant. One patient was 70 years old;

§ , Information of Ebola vaccination status was not documented in six patients with EVD suspicion;

¶ , One EVD patient received the Ebola vaccination 8 days before onset of symptoms (EVD 12; Online Appendix 1);

†† , One of these five patients left the ETC against medical advice. This patient was re-admitted in a critical condition (D5) and died within 24 h after readmission. In this patient, PCR testing revealed Lassa virus and COVID-19 infection;

‡‡ , From the beginning of the outbreak, PCR testing for Ebola virus was available. Viral load results were available several days after start of the outbreak for eight EVD patients. Of note, a high CT value expresses a low VL;

§§ , REGN-EB3 was available from 27 February 2021. Seven EVD patients admitted before this date therefore received the medication several days after ETC arrival. Two EVD patients were too unstable on ETC arrival for REGN-EB3 administration. Two EVD patients passed away before REGN-EB3 was available. One patient survived without receiving REGN-EB3.

All four nonsurviving EVD patients presented with multi-organ dysfunction and died within 48 h of ETC admission (Table 3 and Online Appendix 1^48^). Two of these patients died within 6 h and 13 h after ETC arrival, respectively. Two of the nonsurvivors were 70 years old.

Viral load testing was established several days after declaration of the Ebola outbreak. Therefore, prognostically important VL results at the time of ETC admission^6^ are available in only eight of the 13 EVD patients (Table 3 and Online Appendix 1^48^). Six of these patients survived. All six survivors with available VL results received REGN-EB3. Five of these had low VL (nucleoprotein [NP]-cycle-threshold [CT] > 22). One EVD survivor (EVD patient-8) had a relatively high VL (NP-CT:21). In two of the non-survivors VL results were available (both NP-CT:22).

Ebola-specific monoclonal antibodies were available 2 weeks after the start of the outbreak, explaining delays in administration of REGN-EB3 in seven EVD patients (Table 3 and Online Appendix 1^48^). All eight REGN-EB3 recipients survived, as well as one patient who did not receive REGN-EB3 (EVD Patient 2; Online Appendix 1^48^). Two EVD patients passed away before REGN-EB3 was available. In two EVD patients, vital organ functions could not be stabilised after ETC admission. As administration of monoclonal antibodies can be associated with hypersensitivity reactions and haemodynamic instability,^1,46^ clinical teams aimed to stabilise patients before administration of REGN-EB3.

Mean length of stay (LOS) of EVD patients was 12 days (± 9.7; interquartile range [IQR]: 2–21) versus 2 days (± 1 or [0.9]; IQR 2–3) for patients with suspected EVD. After exclusion of EVD, many of these patients were referred for further care to the regional hospital.

A multidisciplinary ETC team was able to provide essential critical care from the start of the outbreak response, for example, provision of O_2_, fluid management, treatment of acute bacterial co-infections or severe malaria, safe administration of Ebola-specific monoclonal antibodies. Subsequently, a training programme integrated in routine activities supported ETC teams to optimise context-adapted critical care, for example, efficient utilisation of the CUBE;^5^ basic noninvasive respiratory support; management of haemodynamic instability and initiation of low-dose vasopressors; essential management of acute kidney injury (AKI) and electrolyte abnormalities;^10^ and introduction of POCUS.

Transfer of biomedical equipment to N’zérékoré was challenging, and logisticians needed to define priorities (e.g. O_2_ concentrators were transferred before noninvasive respiratory support devices).

Improvement of blood bank services was an initial priority for the ETC laboratory team. Further laboratory services (e.g. biochemistry, haemogram) were established later during the outbreak. Biochemistry results are therefore only available for EVD Patient 13 (Online Appendix 1^48^). However, this example underlines that AKI is a frequent complication in EVD.^4,6^ Laboratory results of this patient show further biochemical derangement: elevated transaminases, amylase, creatinine kinase and low albumin levels.

Importantly, PCR testing of a 23-year-old man with suspected, but nonconfirmed EVD revealed Lassa virus disease and a COVID-19 co-infection (Table 3 and Online Appendix 1^48^).^43^ This patient presented with comparable symptoms to EVD patients. On Day 2, he left the ECT against medical advice. Three days later, he was re-admitted with signs of multi-organ dysfunction and died 24 h later (Online Appendix 1^48^).

### Community engagement

In order to build confidence in outbreak response efforts, communities needed particular support.^12,47^ In this context, family and community members were encouraged to regularly visit ETCs and directly witness the quality of patient care.^5,9^

## Discussion

Implementation of comprehensive Ebola outbreak management allowing early identification of EVD patients, contact tracing and preventive measures (e.g. vaccinations) needs strong community engagement.^1,12,16,47^ Care of patients with suspected and confirmed EVD is best provided close to affected populations to facilitate early initiation of effective treatment and to improve population confidence in outbreak-response measures.^16,47^

During the Ebola outbreak in Guinea (2021), the peripheral ETC in Gouécké could provide essential care. Following prereferral management, critically ill patients with suspected EVD and all confirmed EVD patients were transferred to the ETC in N’zérékoré, where further essential critical care was provided and REGN-EB3 could be administered (Figure 2).^6,10^ Patient referral systems are important elements of critical care^49^ and need to be part of outbreak response preparations.

Nine of the 13 described EVD patients survived. All eight patients who received Ebola-specific monoclonal antibodies (REGN-EB3)^6,46^ survived. All eight REGN-EB3 recipients had stable vital organ functions at the time of drug administration. In the six REGN-EB3 recipients whose VL results were available, five had low VL (NP-CT > 22). One REGN-EB3 recipient with a relatively high VL (NP-CT:21) also survived. The five EVD patients who did not receive REGN-EB3 were either admitted before the medication was available and/or presented with multi-organ dysfunction on ETC arrival (Online Appendix 1^48^). One patient who did not receive REGN-EB3 survived (EVD patient-2; no VL available).

While this series is too small to draw statistically significant conclusions, outcomes are consistent with results of the PALM trial conducted during the Ebola epidemic in the DRC (2018–2020). This trial reported survival above 80% among EVD patients with low VL (NP-CT > 22) benefiting from essential critical care and administration of Zaire ebolavirus-specific monoclonal antibodies.^6^ Results of a WHO-led guideline development group (published in August 2022) recommend the ‘treatment with either MAB114 or REG-EB3 for patients with confirmed Zaire ebolavirus disease and for neonates of unconfirmed EVD status, 7 days or younger, born to mothers with confirmed EVD’.^17,18^ Hence, storage and administration of Ebola-specific monoclonal antibodies need to be part of Ebola outbreak preparedness plans.

Supportive care of EVD patients, outlined in the WHO guidelines, follows general critical care principles aimed at rapid stabilisation of vital organ functions, while considering characteristics of EVD.^3,4,10,19,20,50^

In this series, at least four of the 13 EVD patients needed O_2_ therapy early during ETC admission and may have benefited from noninvasive respiratory support. Over 70% of Ebola patients treated in Europe or the United States required supplemental O_2_, noninvasive or invasive respiratory support during their illness.^4^

Different causes of respiratory dysfunction need to be considered in EVD, for example, acute viral or bacterial co-infections, acute respiratory distress syndrome (ARDS) potentially associated with EVD and/or severe co-infections, fluid overload and cardiac dysfunction.^4,51^ Additionally, the presence of TB and HIV-associated opportunistic infections needs to be evaluated. Different pathologies may co-exist.^4^

Reliable O_2_ supply using O_2_ concentrators and an adequate electricity system were established in the ETC in N’zérékoré following WHO recommendations.^52,53^

Ebola virus disease patients frequently present with haemodynamic instability associated with serious enteral fluid losses and sepsis-related complications,^3,4^ often requiring considerable intravenous fluid replacement. In this context, the use of electrolyte-balanced, isotonic resuscitation fluids may be particularly important.^4,19,20,54^

Training sessions integrated in routine practice enabled the ETC team in N’zérékoré to use basic noninvasive respiratory support as well as low-dose vasopressor infusions.^10^ Additionally, POCUS was introduced to guide haemodynamic stabilisation.^38^ Not all patients could benefit from these interventions (Figure 2 and Online Appendix 1^48^), as training in these elements of essential critical care started in March 2021.

Three of the 13 EVD patients may have benefited from red blood cell (RBC) transfusions (Online Appendix 1^48^). These three patients showed signs of haemorrhage associated with haemodynamic instability and died within 48 h of admission. Arranging an urgent RBC transfusion was challenging at the beginning of the Ebola outbreak, as the ETC was situated several km from the blood bank located in the regional hospital.

Five of the 13 EVD patients and 66% of patients with suspected EVD had positive malaria tests. As all Ebola outbreaks so far have occurred in malaria-endemic regions,^1^ malaria co-infections need to be anticipated as a cause of anaemia in EVD patients.^37^ Importantly, around 6% of patients with suspected EVD (16/276) required RBC transfusions in this study (Table 3).

Haemorrhage is an EVD complication that can occur during advanced stages of the disease process.^3,4,55^ Causes may include disseminated intravascular coagulation and hepatic injury.^3,4^ In this study, clinical signs of coagulopathy associated with haemodynamic instability were seen in three EVD patients. These patients did not survive (Online Appendix 1^48^).

Besides RBC transfusions, options for provision of other blood products should be evaluated in low-resource settings, for example, fresh frozen plasma (FFP) or thrombocyte concentrates.^4^ Fresh dried plasma may be an alternative to provide coagulation factors in settings where production and storage of FFP are challenging.^56^

Developing safe transfusion services was a priority of the ETC laboratory team. Biosecurity measures were established in cooperation with Guinean transfusion services. International and national guidelines for the setup of transfusion services are important elements of outbreak preparedness.

Treatment of neurological emergencies in EVD patients presenting with altered mental status, focal neurological signs or seizures follows essential critical care principles: respiratory and cardiovascular functions need to be stabilised.^4,8,10^ Hypoglycaemia occurs frequently in EVD patients, requiring careful surveillance of blood sugar levels.^3,4,10^

Potential severe co-infections (e.g. bacterial meningitis, severe malaria) need to be treated.^3,4,10,37^ Ebola virus infections can have a direct central nervous system impact, while neurological complications associated with coagulopathies, electrolyte imbalances, renal or hepatic failure need to be considered.^3,4,8^

In the management of status epilepticus refractory to first-line antiseizure medication (ASM), injectable levetiracetam can be of particular benefit in the care of EVD patients or other HIDs. Injectable levetiracetam administration is simple and not associated with severe cardiorespiratory side effects.^24,25,26^ Even when treating EVD patients in CUBEs,^5^ immediate initiation of emergency interventions can be challenging. Additionally, mechanical ventilation is not a feasible option in many resource-limited contexts. Therefore, it may be beneficial to replace ASM with potential cardiorespiratory side effects (e.g. phenobarbitone and phenytoin) with ASM with a better side effect profile.

Acute kidney injury, electrolyte abnormalities as well as acid–base abnormalities are common complications in EVD.^3,4^ Raised creatinine levels are associated with an elevated mortality risk.^4,6,50^ Signs of rhabdomyolysis (elevated creatinine kinase), frequently seen in EVD patients, may contribute to renal dysfunction.^3,4^ This series emphasises the importance of essential AKI management in contexts without ability to initiate renal replacement therapy (Figure 2).^8,10,30^

Elevation of transaminases, amylase and low albumin levels are further biochemistry abnormalities frequently observed in EVD or Lassa virus disease.^3,4,8,10,57^

Malaria co-infections are common among EVD patients treated in malaria-endemic regions and need to be managed urgently.^3,37^ Early initiation of efficient antibiotic treatment for potential bacterial co-infections is equally important.^3,4,6,8,10^

Nutritional support is an essential element in EVD care.^29^ A proactive approach to enteral nutrition in critically ill EVD patients should follow principles of existing international guidelines.^27,28^ Importantly, the WHO has published EVD guidelines for pregnant and breastfeeding women.^34^

Optimised ETC design facilitates provision of optimised supportive care and prevention of cross-infection.^4,5,9^ All patients with suspected or confirmed EVD need to be visible from the low-risk zone, allowing continuous surveillance.^5,9^ In this context, the CUBE provides an innovative interface between health workers positioned in the low-risk zone, patients with confirmed or suspected EVD and clinicians working in the high-risk zone.^5^ Additionally, optimised ETC design facilitates psychological support for children and adults as well as provision of palliative care for patients with end-stage critical illness.^5,9^ Furthermore, the visibility of ETC care promotes trust among populations affected by Ebola epidemics: ‘Newly designed ETCs are not simple isolation units but are treatment units built around needs of patients, staff, families and communities (WHO)’.^5,9^ Experience gained in ETC design can be used in the management of other HID outbreaks.^5,9,57,58,59^ In this context, the WHO and the World Food Program (WFP) initiated a design process for a mobile, rapidly deployable ‘infectious disease treatment module’.^60^

Four of the 13 EVD patients treated in N’zérékoré were health workers, which confirms experience from previous Ebola outbreaks indicating a considerable infection risk during patient care in regular health facilities.^1,61^ In regions potentially affected by Ebola epidemics, health workers therefore need to be vaccinated and safe working environments need to be established.^1,12,61^ Importantly, a recent publication describes postexposure prophylaxis after high-risk contacts using Ebola-specific monoclonal antibodies.^42^

In agreement with larger studies, this series suggests that initial EVD symptoms are similar to early manifestations of common acute infections (e.g. malaria).^3,55,62^ The WHO Ebola case definition therefore has limited specificity, especially in the early phase of a disease outbreak.^4,45,55,62,63^ In this context, screening and triage processes should be strengthened in regular health facilities.^33,59,64^ As signs of EVD or other HIDs may not always be identified during the admission process, HID risk assessment needs to continue during routine inpatient care.

Treatment units with limited bed capacity for patients with suspected HID can be integrated in patient circuits of emergency departments of regular health facilities. Efficient laboratory capacities are needed to guarantee rapid exclusion or confirmation of HIDs.^12,65^ Rapid outbreak response support for these decentralised treatment units could be triggered by the confirmation of HID and/or increasing numbers of patients with suspected HIDs. Rapidly deployable treatment units for HID can be of value in this context.^60,66^

Lassa virus disease was detected in one patient with suspected but nonconfirmed EVD. This patient had clinical and biochemical characteristics similar to EVD patients (Online Appendix 1^48^).^3,4,43^ Acute kidney injury was the predominant organ dysfunction in this patient, accompanied by elevated transaminases and signs of rhabdomyolysis, which then progressed to terminal multi-organ failure (MOF).^43^ It can only be speculated if a COVID-19 co-infection contributed to this patient’s MOF.^43^

Lassa virus disease is endemic in certain regions in West Africa, including Guinea.^67,68,69^ Additionally, in August 2021, the Guinean MOH reported a case of Marburg virus disease (MVD) in the prefecture of the Guéckédou–N’zérékoré region.^44^ These findings emphasise that surveillance and outbreak response systems in West Africa need to prepare for a range of HIDs.^12,65^

Among 681 EVD patients recruited in the PALM trial (DRC; 2018–2019), 25% patients were younger than 18 years and almost 13% younger than 5 years.^6^ In this series, the mean age of EVD patients was around 46 years (Figure 2). It can be speculated that during the Ebola outbreak in Guinea (2021), initially adults were infected, while transmission to a wider spectrum of the population could be prevented by control measures (e.g. vaccinations).^12^

Many patients (38%) with suspected EVD reported in this study were 15 years or younger (17% were 5 years or younger), highlighting that ETCs need to prepare for medical and psychological needs of children and adolescents.^32,35^

One of the 13 described EVD patients was pregnant. She presented 2 days after onset of symptoms with multi-organ dysfunction (NP-CT:22), and despite initiation of essential critical care, she died around 6 h after ETC admission.

Previous reviews report high mortality rates among pregnant EVD patients.^34^ Experiences from the Ebola outbreak in the DRC (2018–2020) emphasise that referral pathways and ETCs need to be prepared for rapid initiation of treatment for pregnant women, including essential critical care, early administration of Ebola-specific monoclonal antibodies,^1,34^ normal and instrumental deliveries, caesarean sections and obstetric emergencies (e.g. postpartum haemorrhage and eclampsia).^34^ Additionally, ETCs need to be set up for essential newborn resuscitation and care.^32,33^

A family accommodation (‘crèche’) needs to be part of ETC design, offering care for children of EVD patients as well as rehabilitation for surviving mothers and children.^9,32,34,35,41^ Furthermore, the WHO recommends programmes for Ebola survivors designed to address medical and psychosocial challenges and to reduce risk of further Ebola virus transmission (e.g. sexual transmission).^34,41^

Health services in Guinea face considerable challenges (e.g. maternal and child health).^70,71^ As a result of the Ebola epidemic in West Africa (2014–2016), a substantial impact on general health services was documented.^72^ Therefore, comprehensive outbreak strategies should support efforts to build resilient health systems in epidemic-affected regions.^72^

### Limitations of the study

Interpretations of study results are limited by the small number of EVD patients. Provision and documentation of clinical care in ETCs is challenging. However, essential critical care could be provided from the start of the outbreak. Further elements of context-adapted critical care, including provision of REGN-EB3 as well as laboratory services, were progressively strengthened. The clinical progress of patients with suspected but subsequently nonconfirmed EVD could only be monitored until discharge from the ETC.

## Conclusion

Multidisciplinary outbreak response teams can rapidly set up well-designed ETCs close to affected populations. Trained clinical teams can provide optimised supportive care, including safe administration of Ebola-specific monoclonal antibodies, as recommended by the WHO. Pragmatic training in essential critical care integrated in routine ETC activities contributed to capacity building.

The results of this series indicate that initial symptoms of EVD are often nonspecific. One patient with suspected EVD had Lassa virus disease, highlighting the importance of considering differential diagnoses during Ebola outbreaks based on regional epidemiology.
